# Protective Effect of Egyptian Propolis against Rabbit Pasteurellosis

**DOI:** 10.1155/2013/163724

**Published:** 2013-08-27

**Authors:** Somia A. Nassar, Amira H. Mohamed, Hamdy Soufy, Soad M. Nasr

**Affiliations:** ^1^Department of Parasitology and Animal Diseases, National Research Center, El-Behouse Street, Dokki, P.O. Box 12622, Giza, Egypt; ^2^Department of Clinical Pathology, Faculty of Veterinary Medicine, Cairo University, P.O. Box 12211, Giza, Egypt

## Abstract

The present study was conducted to study the protective effect of ethanolic extract of propolis given subcutaneously (S/C) either alone or in combination with inactivated formalized *Pasteurella multocida* (*P. multocida*) vaccine in rabbits challenged with virulent *P. multocida* strain. Twenty-eight New-Zealand rabbits, 6–8 weeks old and not vaccinated against pasteurellosis, were randomly divided into four equal groups. Group (1) was kept as nonvaccinated control. Group (2) was injected S/C with propolis. Group (3) was vaccinated (S/C) with *P. multocida* vaccine only. Group (4) was injected with vaccine mixed with propolis as adjuvant. Groups (2, 3, and 4) received the same doses of propolis and vaccine after 4 weeks as a booster dose. The experiment continued for six weeks during which clinical signs, body weight, and mortality rate were recorded. Blood samples were collected every 2 weeks of treatment for evaluating the erythrogram and biochemical parameters. At the end of six weeks, all groups were subjected to challenge with a virulent strain of *P. multocida*. Two weeks later, tissue specimens were collected from different organs for histopathological investigation. Results showed that before challenge all rabbits of different groups were apparently healthy and had good appetite. After challenge, control group (1) showed acute form of the disease, 100% mortality rate, and severe histopathological changes. Rabbits of groups (2 and 3) showed less severe clinical signs, mortality rate, and histopathological changes than control. Rabbits of group (4) were apparently healthy with normal histological picture. In conclusion, an ethanolic extract of propolis injected alone or combined with formalized inactivated *P. multocida* vaccine improved general health conditions, liver and kidney functions in addition to reduction of the severity of adverse clinical signs, mortality rates, and histopathological changes associated with challenge of rabbits with *P. multocida* strain.

## 1. Introduction

One of the most important health problems in rabbit is pasteurellosis, which is considered as a common bacterial disease caused by *Pasteurella multocida* (*P. multocida*) and has been reported as a constant serious and highly contagious disease of domestic rabbits [1]. 

Rabbit pasteurellosis causes symptoms that range from fatal septicemia, severe pleuritis, and pneumonia to less severe sequelae such as multiple abscesses, chronic rhinitis, and otitis media [2]. It mostly affects rabbits at 4–8 weeks of age. Rabbits older than 8 months to 1 year of age showed lower incidence [3]. 

Pasteurellosis exhibited 3 forms in rabbits. The first one is snuffles or nasal catarrhal inflammation which is characterized by acute, subacute, and chronic inflammation of the air passages and lungs. This form of the disease often ends with death and the cured animals became carriers. The second form is characterized by abscess formation at any part of the body and the case is terminated with septicemia. The last form is characterized by genital infection, which manifests as acute and subacute inflammation of uterus and testicles. Also, rhinitis is the most common clinical manifestation in rabbit pasteurellosis [4–6]. 

Propolis is a resinous hive product collected by honey bees from exudates and buds of plants and mixed with wax and bee enzymes [7]. Hegazi et al. [8] recorded that the chemical composition of raw Egyptian propolis sample (collected from Dakahlia Governorate) as investigated by GC/MS, 65 compounds were identified, such as aromatic acids: benzoic, cinnamic, trans-p-coumaric, 3,4-dimethoxycinnamic, ferulic, and caffeic acids. Of the 19 esters identified, Egyptian propolis contained 11 caffeate esters including two new to propolis, tetradecenyl caffeate (isomer) and tetradecanyl caffeate. Egyptian propolis contained some new triterpenoids including lupeol and alpha-amyrin. It also contained flavonoids, sugar, and aliphatic acids. The investigators stated that Dakahlia propolis sample was a typical popular propolis. The composition of the propolis depends upon the time, vegetation, and the area of collection [9].

Propolis has several biological and pharmacological properties, as antimicrobial [10], anti-inflammatory [11], antioxidant [12–15], antiparasitic [16], immune modularity and immune stimulant effects and it increased the percentage of protected animals suggesting its use in vaccines as an adjuvant [17, 18]. 

A reasonable approach to control and eliminate pasteurellosis in rabbits is to develop an improved vaccine as the current experimental vaccines do not provide a complete protection [19].

Therefore, the present work was adopted to evaluate the protective effect of an ethanolic extract of Egyptian propolis when injected S/C alone or in combination with inactivated *P. multocida* vaccine against experimental challenge of rabbits with *P. multocida* strain. The study was based on hematological, biochemical, and histopathological investigations. 

## 2. Materials and Methods

This study was carried out according to guidelines for animal experimentation and approved by the Institutional Animal Care and Use Committee, National Research Centre Animal Care Unit, Dokki, Giza, Egypt.

### 2.1. Animal Used

Twenty-eight male New-Zealand rabbits of 1.5–2 kg body weight and 6–8 weeks old were used in this experiment. Rabbits were not previously vaccinated against pasteurellosis, and bacteriological examination of nasopharyngeal swabs proved that they were free from Pasteurella infection. 

### 2.2. Extraction of Propolis

One hundred grams of the resinous material of Egyptian propolis (obtained from Dakahlia Governorate, Egypt) was cut into small pieces and extracted at room temperature with 50 mL of 70% ethanol. Extraction was performed twice with 24 hours interval. The alcoholic extract was evaporated under vacuum at 50°C until dryness. Obtained dried ethanolic extract of propolis (28 g) was suspended in phosphate buffered saline (PBS) (pH 7.2) [8]. The dose of propolis used in this experiment was 50 mg/kg BW [20]. 

### 2.3. Experimental Design

The experiment was carried out at the Experimental Rabbit Unit of Lab Animal House, National Research Center, Dokki, Giza, Egypt. Rabbits were housed in separate cages, fed on a balanced commercial ration, and water was available *ad libitum*. The animals were assigned into four equal groups which were treated with alcoholic extract of propolis alone or in combination with *P. multocida* inactivated vaccine (obtained from Veterinary Serum and Vaccine Research Institute, Abbasia, Cairo). Propolis was administrated subcutaneously (S/C) (a single dose of 50 mg/kg B.W.). The vaccine was given as a single S/C dose of 2 mL. Treatment of different rabbit groups was as follows: group (1) was injected S/C with 2 mL sterile phosphate buffer saline solution (PBS) and was kept as normal control, group (2) was injected S/C with a single dose of propolis, group (3) was vaccinated with *P. multocida* vaccine only, and group (4) was injected S/C with the vaccine mixed with propolis as an adjuvant. Treatments of propolis and vaccine were repeated as a booster dose after four weeks in all groups. The experiment continued for 6 weeks, at the end of which challenge was performed by injection with virulent strain of *P. multocida. *The strain was obtained from Veterinary Serum and Vaccine Research Institute, Abbasia, Cairo, in the form of lyophilized ampoules. It was activated by culturing in nutrient broth, inoculation in Swiss mice, and reisolation of the organism from heart blood of mice on nutrient agar plates (Difco). Pasteurella colonies were suspended in sterile saline, and the density was adjusted to contain 5 × 10^9^ bacterial cell/mL. The suspension was used for S/C inoculation of rabbits in the challenge test [21].

### 2.4. Erythrogram and Biochemical Analysis

During the 6 weeks experimentation time, rabbits were weighed and blood samples were collected every 2 weeks. Two blood samples were obtained from the ear vein of each rabbit. The first sample was anticoagulated and used for the determination of the erythrocytes count, packed cell volume, hemoglobin concentration, and red cell indices by using Coulter (MEDONIC CA620). The second sample was collected for serum separation and determination of serum biochemical constituents. The activities of aspartate aminotransferase (AST) and alanine aminotransferase (ALT) [22] and alkaline phosphatase [23] were determined. The concentration of urea [24] and creatinine [25] was estimated. Test kits supplied by bioMérieux-France were used. 

### 2.5. Challenge Test and Pathological Studies

At the end of the experiment (6th week), experimental rabbits were challenged by S/C injection of 0.2 mL/rabbit of broth culture of virulent *P. multocida*. Re isolation and identification of *Pasteurella *organisms were done from the heart blood of rabbits died after challenge [21]. Two weeks later after challenge, *Postmortem* findings were detected, and tissue specimens from heart, trachea, lungs, liver, kidneys, and spleen were collected from dead and sacrificed animals, fixed in 10% formol saline, dehydrated, cleared, and embedded in paraffin blocks. Paraffin sections of 5 *μ* thickness were prepared, stained by H&E, and examined microscopically for detection of histopathological alterations [26].

### 2.6. Statistical Analysis

All data were subjected to statistical analysis including the calculation of the mean and standard error. Differences between control and treated groups were tested for significance using a one-way analysis of variance followed by Duncan's multiple range test. Differences were considered significant at *P* < 0.05 level [27] using SPSS version 10 computer programme.

## 3. Results

### 3.1. Clinical Signs

Along the experimental period before challenge, all rabbits of different groups were apparently healthy and had good appetite. During the 1st day after challenge of rabbits with *P. multocida* strain, rabbits of control group (1) showed acute form of the disease (depression, sneezing, and respiratory manifestations), while some of them showed nervous symptoms and sudden death. Rabbits of groups (2) and (3) showed less severe clinical signs than control group. Some rabbits of group (3) showed superficial multiple abscesses as a chronic form of the disease. Rabbits of group (4) were apparently healthy till time of scarification at the end of the experimental (15 days after challenge (dpc)). 

### 3.2. Mortality Rate

No mortalities were observed in group (4) that administrated propolis with vaccine. Mortalities in group (3) (vaccinated only) represented 28.57% after challenge, but in group (2) (administrated propolis only), mortalities were about 57.14%, while mortalities in control group (1) were about 100%. Most of the mortalities occurred during the 2 dpc.

### 3.3. Body Weight

No significant changes were demonstrated in the values of body weight of different experimental rabbit groups along the period of the treatment.

### 3.4. Erythrogram

At the 6th week of treatment, there was a significant decrease in RBCs count and PCV% values, while MCV values exhibited increase in rabbits given vaccine only (group 3) compared to control group (1). Along the period of treatment, no significant changes were demonstrated in the values of Hb content, MCH, and MCHC (Table 1). 

### 3.5. Serum Biochemistry

Compared to control group, the activity of AST and ALT significantly increased at the 2nd and 4th weeks in group (3), while markedly decreased in the other treated groups all over the experimental period. Changes in ALP activity were less marked in different experimental groups (Table 2).

Serum creatinine and urea levels showed significant decrease throughout the experiment in groups (2) and (4). While group (3) demonstrated significant increase in creatinine level all over the experimental time compared to control group (Table 2).

### 3.6. Pathological Findings

#### 3.6.1. Postmortem Examination

Rabbits of control group (1) challenged with *P. multocida* strain showed severe acute form of pasteurellosis. After challenge, rabbits demonstrated severe rhinitis with nasal discharge, congested blood vessels with S/C hemorrhage, presence of blood in thorax and abdomen with severe congestion of trachea, lungs and heart. After 40 hours post challenge rabbits showed congested heart accompanied with enlarged and congested S/C blood vessels, necrotic foci in the liver, brown peritoneum, congested friable kidneys, and dark brown with normal size spleen. Trachea, lungs, and heart were congested, hyperemic and filled with blood. Rabbits administrated propolis only (group 2) showed first deaths after 24 hours after challenge and characterized by sneezing, S/C hyperemic patches, congested heart, trachea and lungs with patches, enlarged and patched liver with necrotic foci, and congested and enlarged spleen. Rabbits administered the vaccine only (group 3) showed less incidence of the disease after challenge represented by presence of multiple lung abscesses, congestion and darkness of the lung and when cut oozing blood, enlarged aorta, and urinary bladder was distended and filled with urine and salts.

Rabbits administered propolis and vaccine (group 4) resisted challenge and were apparently healthy with normal liver, spleen, and heart when scarified at 15 dpc, but some of them showed multiple S/C abscesses in front leg and neck. 

#### 3.6.2. Histopathological Findings

**Figure 1:**
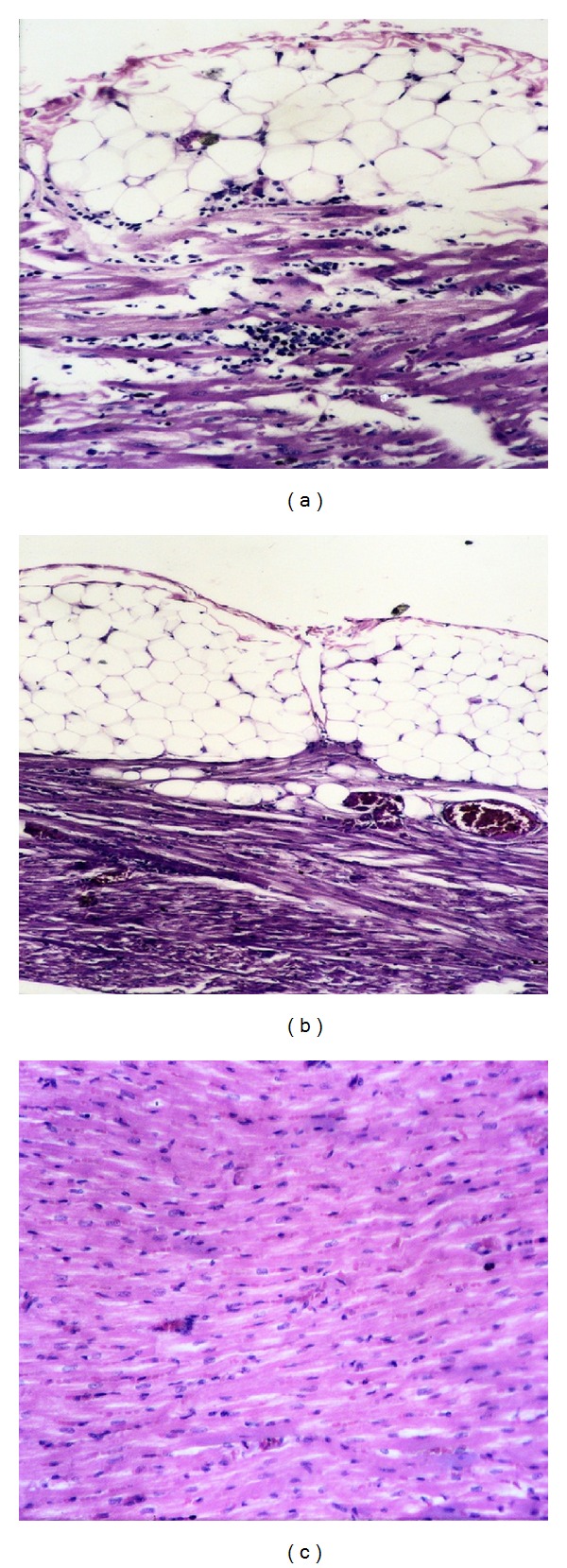
Histopathological changes in the heart of rabbits from different experimental groups. (a) Heart from group (1) rabbits (36 hr pc) showing edema in the pericardium infiltrated with inflammatory cells that extend to the myocardium (H & E ×200). (b) Heart from group (2) rabbits (2 dpc) showing hydropericardium and hemorrhage between the myocardium muscles (H & E ×100). (c) Heart from group (3) rabbits (15 dpc) showing normal myocardial muscle (H & E ×200).

Heart of rabbits from different experimental groups, before challenge, showed normal cardiac tissue. Heart of rabbit from group (1) challenged with *P. multocida* strain, that died within one dpc, showed edema in the pericardium which was infiltrated with inflammatory cells that extended to the myocardium (Figure 1(a)). Heart of rabbit from group (2) that died within 2 dpc showed hydropericardium and hemorrhage between the myocardium muscles (Figure 1(b)). Sections of heart from group (3) and (4) of rabbits, which were sacrificed 15 dpc, showed normal myocardial muscle (Figure 1(c)).

**Figure 2:**
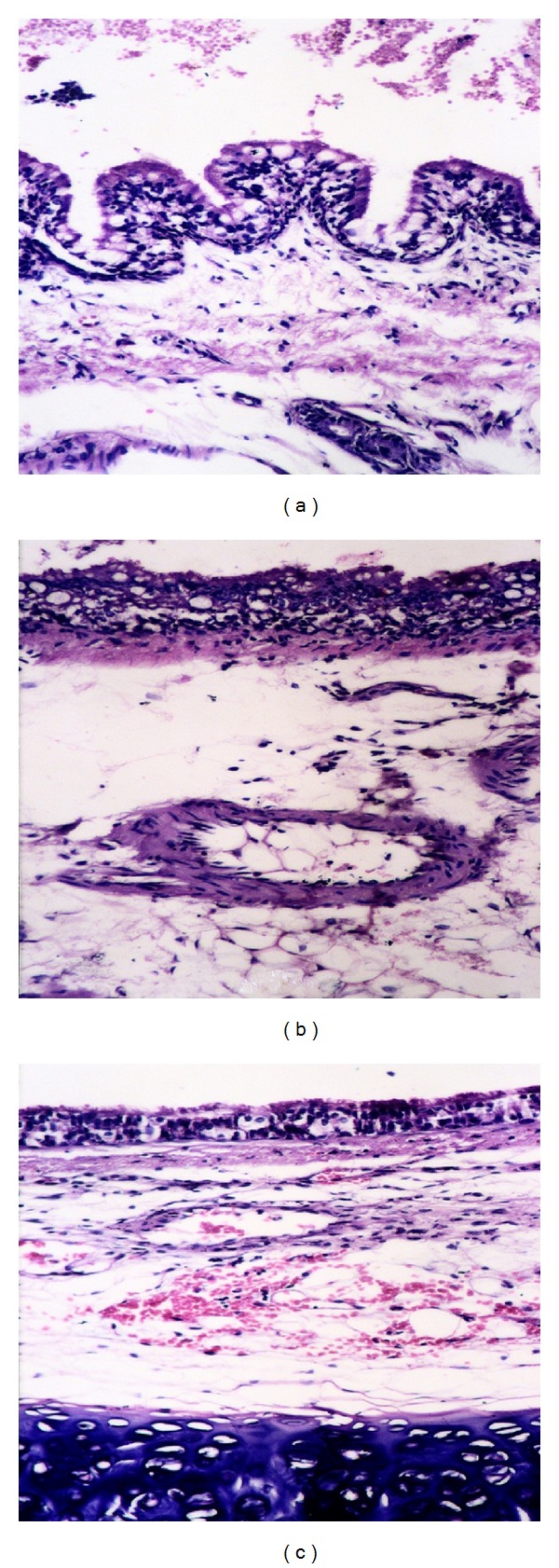
Histopathological changes in the trachea from different experimental groups. (a) Trachea from group (1) rabbits (1 dpc) showing hyperplasia in the lamina epithelialis and leukocytic infiltration in the lamina propia and submucosa. (b) Trachea from group (2) rabbits (2 dpc) showing degenerated mucosa infiltrated with inflammatory cells and edemated submucosa with congestion. (c) Trachea from group (3) rabbits (15 dpc) showing normal mucosa and submucosal edema, hemorrhage, and mononuclear cell infiltration (H & E ×200).

Sections of trachea from rabbits of group (1) which died within 1 dpc showed hyperplasia in the lamina epithelialis and leukocytic infiltration in the lamina propia and submucosa (Figure 2(a)), in addition to marked submucosal edema accompanied with marked congestion of vessels. Trachea from rabbits of group (2), that died 2 dpc, showed degenerated mucosa infiltrated with inflammatory cells and edemated submucosa with congestion (Figure 2(b)). Trachea of rabbits from group (3), which were sacrificed 15 dpc, showed normal mucosa and submucosal edema, hemorrhage, and mononuclear cell infiltration (Figure 2(c)). Trachea of rabbits from group (4), which were sacrificed 15dpc showed normal mucosa with mild edema in the submucosa.

**Figure 3:**
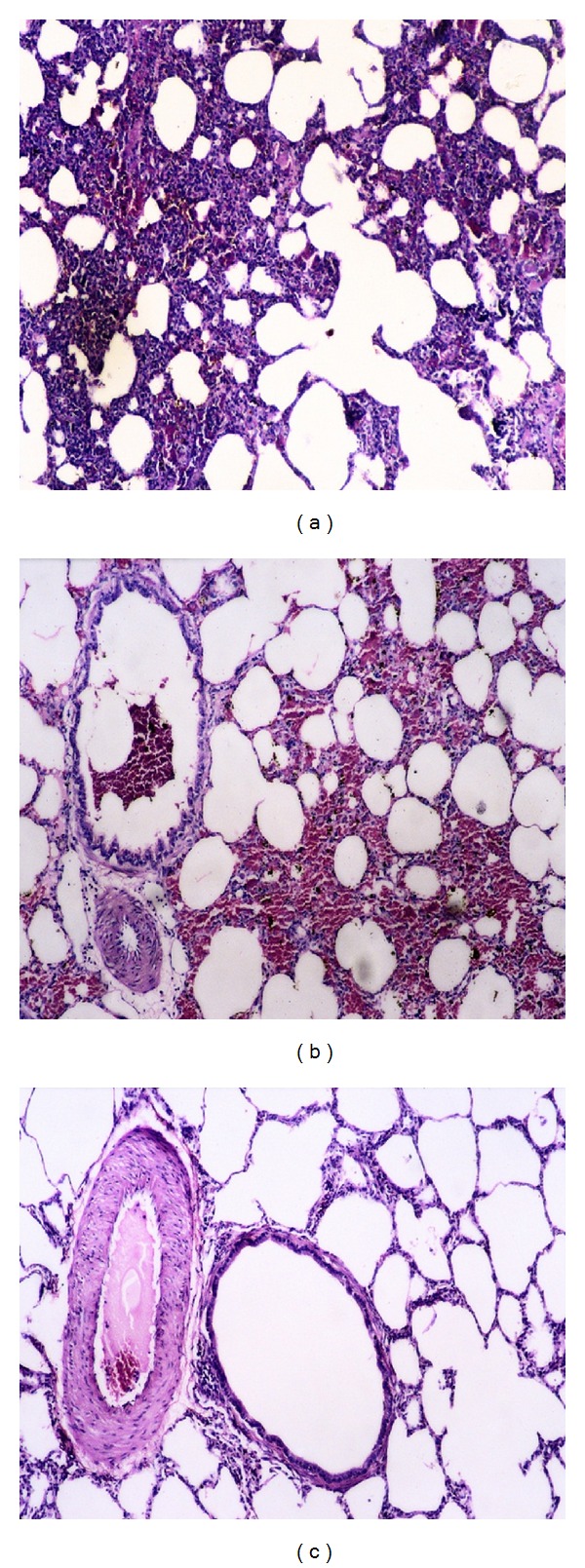
Histopathological changes in the lung from different experimental groups (a) Lung from group (1) rabbits (1 dpc) showing diffused interstitial inflammatory reaction and giant alveoli. (b) Lung from group (2) rabbits (15 dpc) showing chronic venous congestion in the lung tissue. (c) Lung from group (3) rabbits (15 dpc) showing vasculitis and mild interstitial inflammatory reaction (H & E ×100).

Microscopical examination of lung from group (1) before challenge showed normal histological structure. After challenge, the lungs showed peribronchitis, severely congested vessels with vasculitis, and marked alveolar collapse. Moreover, diffused interstitial inflammatory reaction and giant alveoli were observed (Figure 3(a)). Also edema in the plural sac was noticed. Lung of group (2) after challenge showed chronic venous congestion in the lung tissue (Figure 3(b)), while lungs of group (3) showed vasculitis and mild interstitial inflammatory reaction (Figure 3(c)). Sections from lungs of group (4) which were sacrificed 15dpc showed normal lung tissue. 

**Figure 4:**
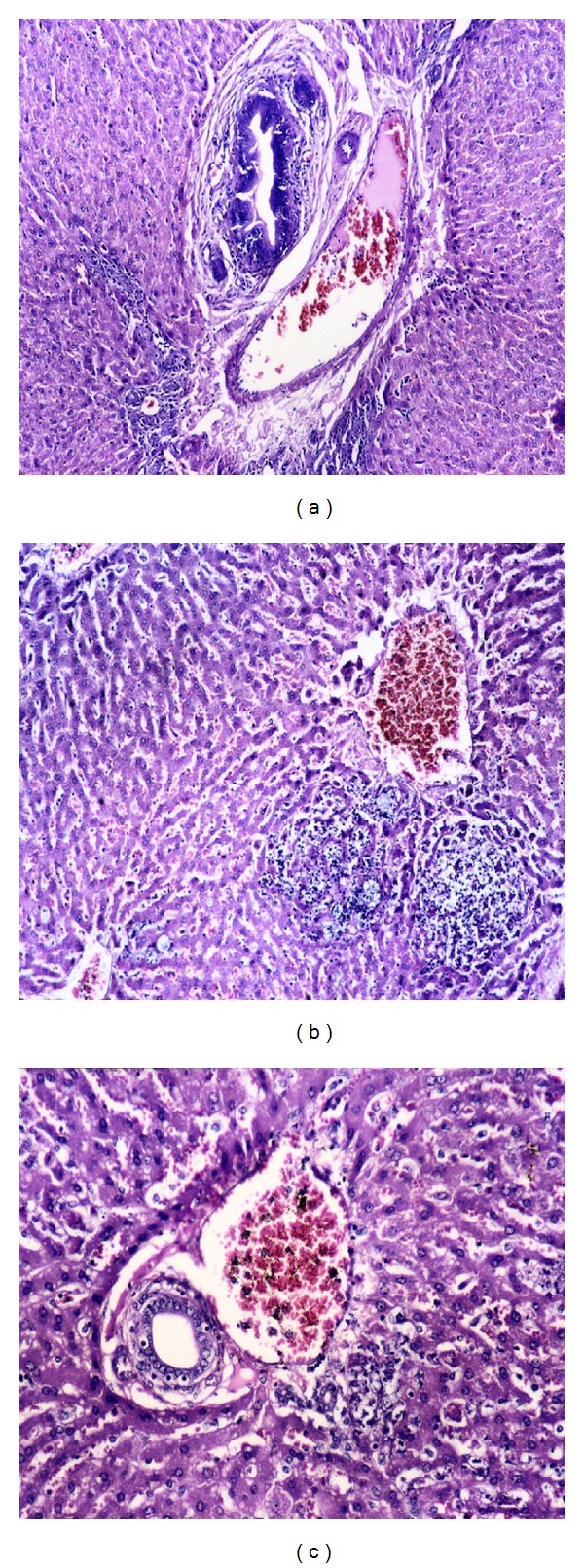
Histopathological changes in the liver from different experimental groups (a) Liver from group (1) rabbits (2 dpc) showing marked portal tract changes in the form of hyperplastic bile duct, congested vessels, newly formed bile ductules, and leukocytic infiltration (H & E ×100). (b) Liver from group (2) rabbits (7 dpc) showing focal scattered necrotic nodules infiltrated with leukocytes (H & E ×100). (c) Liver from group (2) rabbits (15 dpc) showing mild bile duct hyperplasia, mild congestion, and mild inflammatory reaction (H & E ×200).

Microscopical examination of liver from group (1) before challenge revealed normal histology of hepatic lobules. After challenge, the liver showed marked portal tract changes in the form of hyperplasia, congested vessels, newly formed bile ductules, and leukocytic infiltration (Figure 4(a)). Liver of rabbits from group (2), which died after challenge, showed focal scattered necrotic nodules infiltrated with leukocytes (Figure 4(b)) and mild bile duct hyperplasia, mild congestion, and mild inflammatory reaction (Figure 4(c)), but liver of rabbits from group (3) 15 dpc showed normal portal area. Sections from liver of group (4) which were sacrificed 15 dpc showed normal hepatic tissue. 

**Figure 5:**
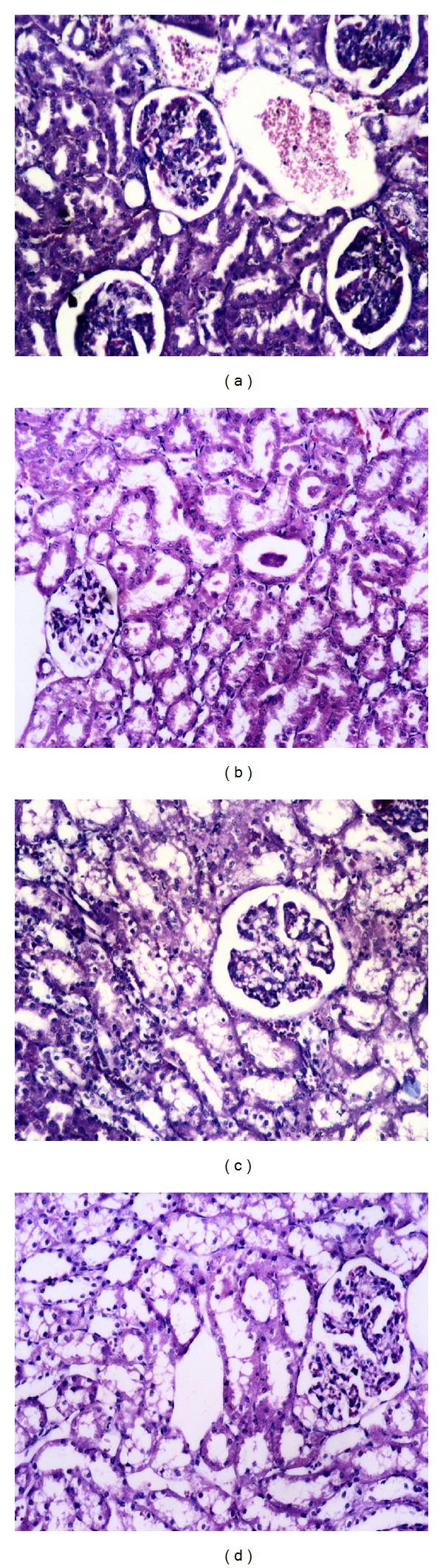
Histopathological changes in the kidney from different experimental groups. (a) Kidney from group (1) rabbits (1 dpc) showing congestion in the interstitial blood vessels and glomerular capillaries. (b) Kidney from group (1) rabbits (2 dpc) showing degenerated tubules with formation of renal hyaline casts. (c) Kidney from group (2) rabbits (15 dpc) showing severe vacuolation in the tubules and glomerular tuft. (d) Kidney from group (3) rabbits (15 dpc) showing mild vacuolation in the tubules and glomerular tuft (H & E ×200).

Microscopical examination of kidney from group (1) before challenge showed normal renal tissue, while after challenge the kidney showed congestion in the interstitial blood vessels and glomerular capillaries (Figure 5(a)), in addition to degenerated tubules with formation of renal hyaline cast (Figure 5(b)). Kidneys from group (2) after challenge showed severe vacuolation in the tubules and glomerular tuft (Figure 5(c)). Kidneys from group (3) showed mild vacculation in the tubules and glomerular tafft (Figure 5(d)). Kidneys from group (4) which were sacrificed 15 dpc showed normal renal tissue.

**Figure 6:**
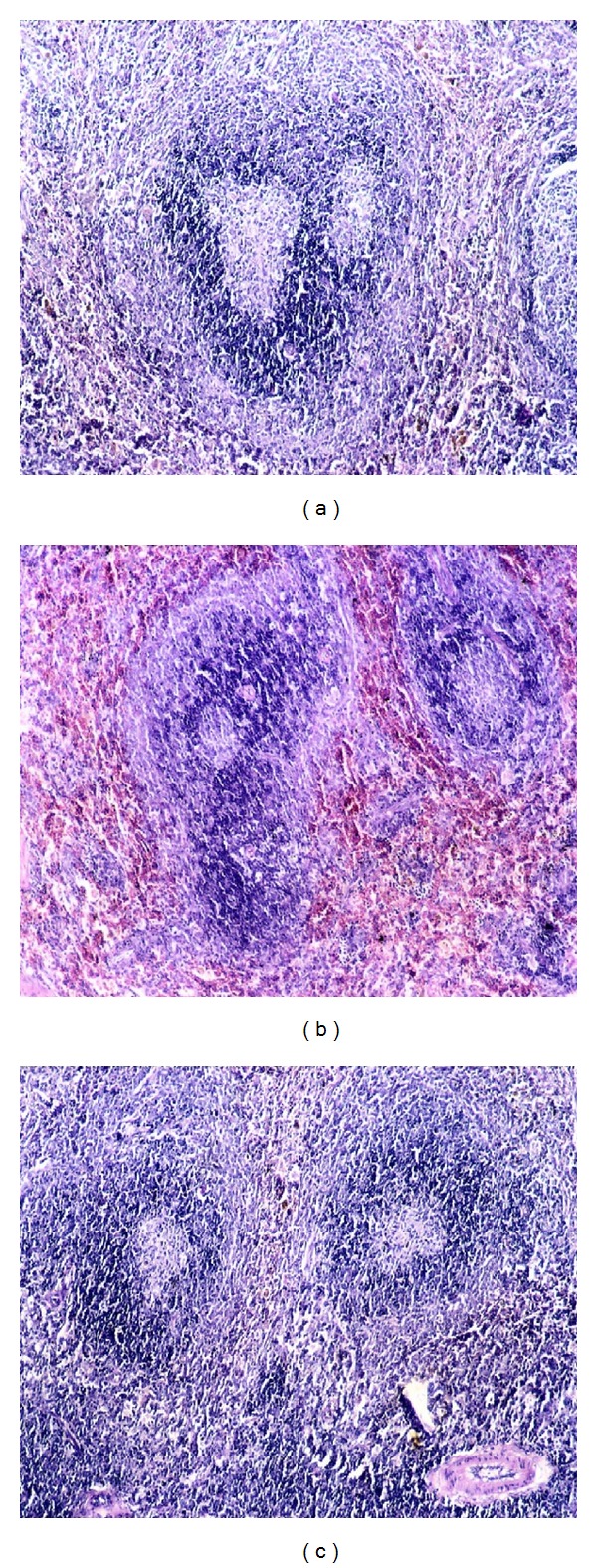
Histopathological changes in the spleen from different experimental groups. (a) Spleen from group (1) rabbits (2 dpc) showing depletion in the lymphoid follicles. (b) Spleen from group (2) rabbits (2 dpc) showing hemorrhage between the lymphoid follicles. (c) Spleen from group (3) rabbits (15 dpc) showing atrophied follicles (H & E ×100).

Microscopical examination of spleen from group (1) before challenge showed normal lymphoid follicles; in contrast after challenge spleen showed depletion in the lymphoid follicles (Figure 6(a)). In group (2), there was hemorrhage in between the lymphoid follicles at 2 dpc (Figure 6(b)). Spleen from group (3) showed atrophied follicles (Figure 6(c)). Spleen from group (4) which was sacrificed 15 dpc showed normal splenic follicles.

## 4. Discussion

The present experiment was conducted to study the effect of an ethanolic extract of propolis given by S/C injection either alone or in combination with inactivated formalized *P. multocida* vaccine on general performance, erythrogram, biochemical parameters, and pathological lesions induced by challenge of rabbits with virulent *P. multocida* strain at the end of the experiment.

The symptoms which were observed within one dpc of group (1) with *P. multocida* strain ranged from severe acute to subacute forms of the disease. Signs of septicemia, acute rhinitis, sneezing, bronchopneumonia, and conjunctivitis and abscess formation agreed with that reported by [1, 2, 28]. These signs were confirmed by macroscopic and microscopic examination of different organs of rabbits of group (1) after challenge. Less severe symptoms observed in group (2) may be due to the effect of propolis as antibacterial, anti-inflammatory, and immunomodularity agent and increase the antibody production [19, 29, 30]. In addition, propolis has broad activities against *P. multocida in vitro *and *in vivo* (in the tracheal region of the rabbits) [31]. The chronic form of pasteurellosis, superficial multiple abscesses observed in group (3) may be due to that inactivated vaccine enhance mainly humeral immunity response and immunoglobulins (Ig) level specially IgG [19, 32, 33]. These results were confirmed by the normal microscopic structure of spleen tissue after challenge. Absence of clinical signs in group (4) may be attributed to the synergetic protective effect of both propolis and vaccine. These results agreed with the normal histological structure of different organs in this group.

Mortality rate was 100% in group (1), 57.14% in group (2), and about 28.57% in group (3). Mortality in group (1) may be due to severe septicemia and bronchopneumonia [5, 34], while lower mortalities in group (2) may be attributed to the antibacterial, anti-inflammatory, immune stimulant, and immune modularity effects of propolis [19, 35]. Good protection in group (3) may be attributed to good antibody response and protection induced by the vaccine against experimental challenge with *P. multocida* [19, 36, 37].

Results of body weight showed no significant difference between all groups along the experimental period. 

Results of erythrogram revealed significant decrease in RBCs count, PCV in group (3) which may be due to the cytotoxic effect of vaccine causing inhibition of erythropoiesis [38]. These results agreed with [39].

Regarding serum enzyme activities, AST showed significant gradual decrease in different groups along the period of experiment except group (3) in comparison with control group. This result agreed with Talas and Gulhan [40]. Administration of propolis to rats at a dose of 150–1500 mg/kg BW caused slight inhibition in the activity of aminotransferase enzymes [41]. The demonstrated result in the present study revealed that administration of propolis had no toxic effect on rabbit. A significant decrease in serum ALT level was observed in propolis-treated groups; similar result was observed by Eraslan et al. [42]. No alterations in the activity of AST and ALT in serum of rabbits treated with crude propolis extract [43]. The level of ALP was decreased in group administrated propolis compared to the control group. This decrease may be due to the action of propolis as reducing agent to ALP. These results agreed with Eraslan et al. [42]. Results of liver enzymes activities support that propolis is able to induce hepatoprotective effects which are similar to those of the previous work on propolis that act as hepatoprotective against d-galactosamine, and paracetamol induced liver damage in rats and mice, respectively [44, 45]. The present results were confirmed by absence of histopathological changes in livers of groups (2) and (4) in comparison with liver of control group. 

Results of serum creatinine and urea revealed significant decrease in groups (2 and 4) compared to control group. Similar results were recorded by Sforcin et al. [46]. They found that propolis did not induce kidney damage in rats as demonstrated by normal levels of urea and creatinine. 

## 5. Conclusion

ethanolic extract of propolis injected S/C alone or combined with formalized inactivated *P. multocida *vaccine improved general health conditions, liver and kidney functions in addition to reduction of the severity of adverse clinical signs, mortality rates, and histopathological changes associated with challenge of rabbits with *P. multocida strain*.

## Figures and Tables

**Table 1:** Erythrogram in different experimental groups of rabbits before and after treatment for six weeks (Mean ± SE, *N* = 5).

Parameters	Periods (week)	Groups				Sig.
		G1 Control	G2 Propolis S/C	G3 Vaccine S/C only	G4 Propolis + Vaccine S/C	
RBCs count (×10^6^/*µ*L)	0	5.73 ± 0.31	5.73 ± 0.31	5.47 ± 0.35	5.27 ± 0.21	NS
	2	5.38 ± 0.16	5.56 ± 0.17	5.16 ± 0.14	5.46 ± 0.34	NS
	4	5.84 ± 0.33	5.75 ± 0.28	5.04 ± 0.16	5.81 ± 0.33	NS
	6	6.35 ± 0.18^a^	5.42 ± 0.25^ab^	4.93 ± 0.45^b^	5.42 ± 0.20^ab^	∗

Packed cell volume (PCV) (%)	0	34.24 ± 1.82	34.24 ± 1.82	33.84 ± 1.89	30.17 ± 0.90	NS
	2	32.30 ± 0.90	33.12 ± 0.52	30.48 ± 0.78	30.92 ± 1.63	NS
	4	32.54 ± 1.54	34.36 ± 1.68	31.25 ± 1.27	33.84 ± 1.69	NS
	6	34.55 ± 0.53^a^	33.45 ± 1.16^a^	29.64 ± 2.51^c^	31.28 ± 1.37^b^	∗

Hemoglobin (g/dL)	0	11.78 ± 0.67	11.78 ± 0.67	10.92 ± 0.56	10.37 ± 0.31	NS
	2	11.24 ± 0.30	11.32 ± 0.15	10.58 ± 0.30	10.84 ± 0.60	NS
	4	10.88 ± 0.47	11.46 ± 0.60	10.40 ± 0.46	11.26 ± 0.41	NS
	6	11.60 ± 0.18	11.33 ± 0.34	10.24 ± 0.91	11.74 ± 0.57	NS

Mean corpuscular volume (MCV) (fl)	0	59.80 ± 0.82	59.80 ± 0.82	63.80 ± 3.36	57.47 ± 0.72	NS
	2	60.12 ± 0.95	59.66 ± 1.58	59.13 ± 1.23	56.78 ± 1.10	NS
	4	59.94 ± 0.92^b^	59.74 ± 0.77^b^	61.98 ± 1.04^a^	58.32 ± 1.20^b^	∗
	6	58.63 ± 2.20^b^	59.28 ± 1.41^b^	62.18 ± 0.78^a^	60.08 ± 1.37^b^	∗

Mean corpuscular hemoglobin (MCH) (pg)	0	20.52 ± 0.15	20.52 ± 0.15	20.60 ± 0.56	19.73 ± 0.21	NS
	2	20.90 ± 0.26	20.38 ± 0.32	20.55 ± 0.40	19.94 ± 0.37	NS
	4	20.10 ± 0.46	19.96 ± 0.33	20.60 ± 0.31	19.48 ± 0.85	NS
	6	19.35 ± 0.35	20.08 ± 0.55	20.88 ± 0.24	20.71 ± 0.84	NS

Mean corpuscular hemoglobin concentration (MCHC) (g/dL)	0	34.36 ± 0.37	34.36 ± 0.37	32.46 ± 1.00	34.30 ± 0.22	NS
	2	34.82 ± 0.36	36.04 ± 1.86	34.73 ± 0.14	35.08 ± 0.17	NS
	4	33.52 ± 0.33	33.38 ± 0.21	33.25 ± 0.47	33.38 ± 0.84	NS
	6	33.57 ± 0.07	33.88 ± 0.22	34.49 ± 0.18	34.24± 0.40^a^	NS

Means with different superscripts in the same row are significantly different at *P* < 0.05. *P < 0.05, NS: non-significant.

**Table 2:** Serum enzymes activities and serum creatinine and urea levels in different experimental groups of rabbits before and after treatment for six weeks (Mean ± SE, *N* = 5).

Parameters	Periods (week)	Groups				Sig.
		G1 Control	G2 Propolis S/C	G3 Vaccine S/C only	G4 Propolis + vaccine S/C	
Aspartate amino transferase (IU/L)	0	48.46 ± 2.40	48.69 ± 0.36	47.39 ± 1.20	49.42 ± 2.90	NS
	2	49.71 ± 2.94^b^	40.96 ± 0.46^c^	60.73 ± 2.29^a^	49.11 ± 0.74^b^	∗
	4	48.90 ± 0.01^b^	44.41 ± 1.00^c^	66.86 ± 2.40^a^	43.45 ± 0.49^c^	∗
	6	51.26 ± 1.87^a^	43.13 ± 1.47^c^	50.35 ± 1.90^ab^	41.16 ± 0.83^c^	∗

Alanine amino transferase (IU/L)	0	54.79 ± 0.82	54.90 ± 3.36	55.70 ± 1.68	56.37 ± 3.26	NS
	2	51.91 ± 0.46^b^	47.60 ± 4.23^c^	60.45 ± 0.57^a^	45.40 ± 3.80^c^	∗
	4	41.24 ± 3.94^c^	47.47 ± 3.09^b^	55.68 ± 1.24^a^	39.28 ± 1.50^c^	∗
	6	53.07 ± 1.23^a^	40.27 ± 1.91^c^	47.25 ± 2.75^b^	39.28 ± 1.50^c^	∗

Alkaline phosphatase (IU/L)	0	15.74 ± 1.23	15.59 ± 2.06	16.67 ± 1.09	16.46 ± 0.17	NS
	2	16.87 ± 1.02^a^	15.03 ± 0.29^ab^	17.59 ± 0.70^a^	16.39 ± 1.11^a^	NS
	4	15.55 ± 1.35^b^	14.87 ± 1.55^b^	20.14 ± 1.17^a^	15.91 ± 0.90^b^	∗
	6	15.15 ± 0.83	13.43 ± 0.94	15.83 ± 1.00	14.49 ± 0.52	NS

Creatinine (mg/dL)	0	1.37 ± 0.05	1.68 ± 0.04	1.80 ± 0.12	1.68 ± 0.07	NS
	2	1.17 ± 0.08^b^	0.91 ± 0.04^b^	1.42 ± 0.01^a^	0.87 ± 0.04^b^	∗
	4	1.07 ± 0.03^b^	0.76 ± 0.03^c^	1.39 ± 0.04^a^	0.76 ± 0.05^c^	∗
	6	1.22± 0.27^a^	0.61 ± 0.03^b^	1.24 ± 0.02^a^	0.60 ± 0.03^b^	∗

Urea (mg/dL)	0	35.56 ± 1.86	35.60 ± 1.81	33.22 ± 1.47	36.13 ± 2.09	NS
	2	39.02 ± 1.54^a^	27.14 ± 1.54^d^	35.37 ± 0.97^b^	31.11 ± 1.42^c^	∗
	4	39.85 ± 2.21^a^	24.29 ± 1.06^c^	32.75 ± 1.67^b^	29.94 ± 1.22^b^	∗
	6	38.15 ± 1.49^a^	24.23 ± 1.05^c^	40.14 ± 0.82^a^	33.25 ± 1.43^b^	∗

Means with different superscripts in the same row are significantly different at *P* < 0.05. *P < 0.05. NS: nonsignificant.
